# The bright side of words: Norms for 9000 Spanish words in seven discrete positive emotions

**DOI:** 10.3758/s13428-023-02229-8

**Published:** 2023-09-25

**Authors:** José A. Hinojosa, Marc Guasch, Pedro R. Montoro, Jacobo Albert, Isabel Fraga, Pilar Ferré

**Affiliations:** 1 Instituto Pluridisciplinar, Universidad Complutense de Madrid, Madrid, Spain; 2 Dpto. Psicología Experimental, Procesos Cognitivos y Logopedia, Universidad Complutense de Madrid, Madrid, Spain; 3 Centro de Investigación Nebrija en Cognición (CINC), Universidad Nebrija, Madrid, Spain; 4 Department of Psychology and CRAMC, Universitat Rovira i Virgili, Tarragona, Spain; 5 Departamento de Psicología Básica 1, Facultad de Psicología, Universidad Nacional de Educación a Distancia (UNED), Madrid, Spain; 6 Departamento de Psicología Biológica y de la Salud, Facultad de Psicología, Universidad Autónoma de Madrid, Madrid, Spain; 7 Cognitive Processes & Behaviour Research Group, Department of Social Psychology, Basic Psychology & Methodology, University of Santiago de Compostela, Santiago de Compostela, Spain

**Keywords:** Positive emotions, Emotional ratings, Awe, Contentment, Amusement, Excitement, Serenity, Relief and pleasure

## Abstract

In recent years, assumptions about the existence of a single construct of happiness that accounts for all positive emotions have been questioned. Instead, several discrete positive emotions with their own neurobiological and psychological mechanisms have been proposed. Of note, the effects of positive emotions on language processing are not yet properly understood. Here we provide a database for a large set of 9000 Spanish words scored by 3437 participants in the positive emotions of awe, contentment, amusement, excitement, serenity, relief, and pleasure. We also report significant correlations between discrete positive emotions and several affective (e.g., valence, arousal, happiness, negative discrete emotions) and lexico-semantic (e.g., frequency of use, familiarity, concreteness, age of acquisition) characteristics of words. Finally, we analyze differences between words conveying a single emotion (“pure” emotion words) and those denoting more than one emotion (“mixed” emotion words). This study will provide researchers a rich source of information to do research that contributes to expanding the current knowledge on the role of positive emotions in language. The norms are available at https://doi.org/10.6084/m9.figshare.21533571.v2

## Introduction

Studies investigating the processing of emotional language have shown that emotion permeates all aspects of language (see Citron et al., 2012, and Hinojosa et al., 2020, for reviews), including morphology (Hinojosa et al., 2022; Kuperman, 2013; Lapesa et al., 2017), phonology (Adelman et al., 2018; Conrad et al., 2022; Schmidtke & Conrad, 2018), semantics (Haro et al., 2022; Herbert et al., 2008; Kissler et al., 2009), grammar (Fraga et al., 2021; Hatzidaki & Santesteban, 2022; Poch et al., 2022), or pragmatics (Aguado et al., 2019; Schindler et al., 2019), from early development stages of a child's life (Grosse et al., 2021; Sabater et al., 2020, 2022). Most work followed the dimensional approach to emotions (Russell, 2003). According to this view, the two core dimensions of valence (the hedonic tone of a word referent, ranging from negative/unpleasant to positive pleasant) and arousal (the degree of activation elicited by a word referent, ranging from calming to exciting) account for most emotional meaning of words. This approach has been very productive for emotional language research. For instance, the results of several studies across different languages such as English, German, or Chinese have shown a delayed processing of negative words relative to neutral words (Estes & Adelman, 2008; Kuperman et al., 2014; Yao et al., 2016). In contrast, a processing advantage for positive over neutral words has typically been observed (Rodríguez-Ferreiro & Davies 2019; Vinson et al., 2014; Yap & Seow, 2014). These effects are sometimes mediated by an interaction between valence and arousal. In this vein, positive low-arousal words and negative high-arousal words elicit speeded processing and enhanced brain activation compared to positive high-arousal words and negative low-arousal words (Citron et al., 2014; Hofman et al., 2009). An alternative account conceives the existence of a limited number of discrete primary categories of emotion that have been shaped during the evolution to serve different adaptive functions through specific neural signatures, face expressions, cognitive appraisals, and behavioral action tendencies (Ekman, 1992, 1993). These basic emotions typically include happiness, anger, fear, sadness, disgust, and surprise. Although some objections have been formulated to the basic emotions model^1^, this theoretical framework has inspired some work investigating the processing of emotional words. The results of these studies showed delayed identification of German disgust-related words (but not of fear-related words) relative to neutral words (Briesemeister et al., 2011a), increased accuracy in recognizing German words that convey fear compared to neutral words (Briesemeister et al., 2011b), a lower percentage of errors to Spanish disgust-related words than to fear-related words (Ferré et al., 2018), enhanced activation in the insula to disgust-related compared to neutral French words (Ponz et al., 2014), or slower responses (Huete-Pérez et al., 2019) and increased activity in the gamma band (Santaniello et al., 2022) to Spanish fear words relative to anger words.

Of note, early formulations of discrete emotions theories assumed the existence of several basic negative emotions like fear, anger, disgust, or sadness, while positive emotions were mainly represented by the single construct of happiness or joy (Ekman, 1992; Tomkins, 1984). This broad conception of positive emotions was later criticized, and subsequent classifications included other positive emotions such as love, relief, excitement, or awe (e.g., Fehr & Russell, 1984; Roseman et al., 1990), although these taxonomies were still outnumbered by negative emotions. More recently, some authors have emphasized the need of further differentiating between discrete positive emotions (Cordaro et al., 2020; Shiota et al., 2017), and the theoretical landscape has shifted to conceptualize positive emotions as a family of specific states that emerged from a common ancestor facilitating an adaptive response to particular kinds of fitness-relevant resources (Shiota et al., 2017). In agreement with this view, results from a growing number of studies in the field of affective science have led to the characterization of several positive emotions as separate functional entities with their own idiosyncratic behavioral, psychological, and neurobiological markers, such as amusement, serenity, pride, love, awe, relief, compassion, contentment, gratitude, pleasure, excitement, or hope (see Campos et al., 2013; Keltner & Cowen, 2021; Saarimaki et al., 2018; Sauter, 2017; Shiota et al., 2017; Van Cappellen et al., 2023; Warren et al., 2021, for a synthesis of differences in evolution, expressive behavior, and neurophysiology).

Just to give a few examples, differences in nonverbal cues during communication have been reported. In this sense, contentment is associated with vocalizations of long durations and low-intensity smiles, while amusement triggers large smiles with open jaw and head movements. Relief elicits sighs and smiles with eyelids tightened, awe gives rise to inhalations and widened eyes, and compassion evokes patting and stroking (e.g., Haidt & Keltner, 1999; Krumhuber & Scherer, 2011; Sauter et al., 2010; Shiota et al., 2003). Also, using various positive-emotion elicitation paradigms, several studies have reported that contentment evokes a physiological response characterized by a higher sympathetically deactivating activity (e.g., decreased heart-rate and electrodermal activation) compared to amusement (increased skin conductance, heart-rate variability, and respiratory activity). Pleasure or liking is associated with respiratory variability, increased cardiac vagal control, and increased finger temperature, whereas relief is associated with moderate cardiovascular changes and decreased electrodermal reactivity. Finally, pride gives rise to unchanged total peripheral resistance and decreased heart rate (e.g., Bradley et al., 2008; Cristie & Friedman, 2004; Herrald & Tomaka, 2002; Gruber et al., 2008; Palomba et al., 2000). Regarding the neural correlates of different positive emotions, prior reports have shown a reduced engagement in self-referential processing evidenced by decreased activation in the frontal pole and the posterior cingulate cortex while participants experienced awe. In contrast, enhanced activation of the frontal pole has been linked to the feeling of hope, while increased activity in the anterior cingulate cortex is elicited by relief (Navratilova et al., 2015; van Elk et al., 2019; Wang et al., 2017).

Recently, Weidman and Tracy (2020) proposed a taxonomy of positive emotions based on the analysis of language used to describe feelings, thoughts, and behaviors in English that included nine states (i.e., temporary feelings: awe, amusement, interest, authentic pride, hubristic pride, compassion, gratitude, hope, love) and five traits (i.e., more enduring feelings: hope, love, amusement, authentic pride, hubristic pride) positive emotions. Also, Mikels et al. (2005) provided descriptive categorical data of linguistic terms for a set of positive pictures from the International Affective Picture System (IAPS, Lang et al., 1999) that included labels such as awe, amusement, excitement, or content. Finally, Malik and Hussain (2017) analyzed e-commerce websites to find the best predictors for helpfulness of online reviews. They observed that the discrete positive emotions of trust, joy, and anticipation made the greatest contribution to perceived review helpfulness. Overall, these findings suggest that there might be a close relationship between discrete positive emotions and different linguistic processes, and leaves open an avenue to examine questions about the role of positive emotions in lexico-semantic and morphosyntactic processing, or the representation of distinct positive emotions in semantic memory.

The lack of normative studies is a barrier that at least partially has precluded the design and development of studies with a focus on the interplay between discrete positive emotions and linguistic processes. In this sense, there are some large emotional lexicon projects that have collected data for 20,007 English words in the dimensions of valence, arousal, and dominance (NRC VAD Lexicon; Mohammad, 2018), and for 14,182 words in the basic emotions of anger, disgust, fear, joy, sadness, surprise, trust, and anticipation (NRC Emotion Lexicon; Mohammad & Turney, 2013). These datasets also provide automatic translations from English words in over 100 languages. Nonetheless, most efforts have concentrated on collecting data for hundreds or thousands of words in the dimensions of valence and arousal (e.g., Chinese: Yao et al., 2017, Xu et al., 2022; Croatian: Ćoso et al., 2019; Dutch: Moors et al., 2013; English: Bradley & Lang, 1999, Warriner et al., 2013; Finnish: Söderholm et al., 2013; French: Monnier & Syssau, 2014; German: Citron et al., 2020; Kanske & Kotz, 2010; Indonesian: Sianipar et al., 2016; Italian: Montefinese et al., 2014; Polish: Imbir, 2015; Portuguese: Soares et al., 2012; Spanish,: Guasch et al., 2016, Stadthagen-González et al., 2017), or in the primary emotions of happiness, anger, fear, disgust, sadness, or surprise (e.g., Croatian: Ćoso et al., 2022; English: Stevenson et al., 2007; German: Briesemeister et al. 2011b; Spanish: Ferré et al., 2017, Stadhagen-González et al., 2018; Turkish: Kapucu et al., 2021). A high consistency of affective norms has been reported across languages (e.g., between Croatian and both Spanish and English, Ćoso et al., 2019; or between Italian and both English and Spanish, Montefinese et al., 2014). Of note, to the best of our knowledge, there is just a single study that reported norms for 1031 French words for the five discrete positive emotions of awe, amusement, contentment, excitement, and serenity (Syssau et al., 2021). The results of multidimensional scaling analyses showed that a number of words were specifically related to each of these positive emotions, which argues in favor of the importance of differentiating positive emotions beyond happiness (Shiota et al., 2017; Syssau et al., 2021).

In the current study, we aim at collecting ratings (i.e., judgements about the emotions conveyed by words) for a large set of 9000 Spanish words in seven discrete positive emotions. Our selection was based in current taxonomies of positive emotions (Shiota et al., 2017) and prior normative studies with French words (Syssau et al., 2021), as well as in scores of emotional prototypicality in both English and Spanish (i.e., the degree to which a word represents an emotion; Fehr & Russell, 1984; Pérez-Sánchez et al., 2021). Thus, the main focus was on the five positive emotions examined by Syssau et al. (2021): amusement, which is related to play, joy, or perceived funniness, and elicits low urge to approach compared to other high-approach positive emotions like awe (Campbell et al., 2022; Martin & Ford, 2018; Warren et al., 2021); awe, which is an emotion triggered by vast, unfamiliar stimuli that cannot be accounted for by one’s stored knowledge, and arises cognitive accommodation processes linked to the revision of current frames of reference to interpret these stimuli (Danvers & Shiota, 2017; Gottlieb et al., 2018; Keltner & Haidt, 2003; Piff et al., 2015); contentment, which is associated with perceived goal attainment, a sense of completeness and peaceful acceptance of current circumstances, and possibly reflects an assessment of the social and psychological resources that we have produced to sustain global wellness (Berenbaum et al., 2019; Chua et al., 2022; Cordaro et al., 2016); excitement or anticipatory enthusiasm, which is a high arousing positive emotion elicited by events or stimuli that are novel, interesting, and challenging, and promotes attention to and acquisition of rewards (Griskevicius et al., 2010; Shiota et al., 2011); and serenity, which refers to sustained inner strength and peace that is characterized by a feeling of secular spirituality and the ability to enjoy present experience, and has a buffer function in ameliorating stress and promoting mental well-being (Roberts & Cunningham, 1990; Soysa et al., 2021; Wolfradt et al., 2014). Additionally, we collected for the first time ratings for two other discrete positive emotions that have been included in several taxonomies of positive emotions (Keltner & Cowen, 2021; Shiota et al., 2017; Weidman & Tracy, 2020): pleasure or liking^2^, which is an affective reaction linked to both subjective and objective assessments of current or expected hedonic reactions, and it is thought to play a relevant role in reward learning based on incentive salience of stimuli (Berridge & Kringelbach, 2013; Sharot et al., 2009); and relief, which refers to a positive feeling elicited by the absence of an anticipated threat or the cessation of negative stimulation that results in reduction of distress (Deutsch et al., 2015; Kreibig, 2010; San Martín et al., 2020).

A second goal of our study was to examine the relationship between scores in these positive emotions, and those for affective dimensions and basic emotions from prior normative studies in Spanish (Ferré et al., 2017; Hinojosa et al., 2016a; Stadhagen-González et al., 2017, 2018). Additionally, since prior evidence coming from several languages like English, Italian, Croatian, Dutch, Spanish, or French has shown a close link between both dimensional and discrete emotional features of words (Ćoso et al., 2022; Stadhagen-González et al., 2017; Syssau et al., 2021), and several lexico-semantic properties such as word frequency (e.g., Méndez-Bértolo et al., 2011; Montefinese et al., 2014; Scott et al., 2009), familiarity (e.g., Hinojosa et al., 2016b; Warriner et al., 2013), age of acquisition (e.g., Ćoso et al., 2022; Moors et al., 2013; Ponari et al., 2018), concreteness (e.g., Hinojosa et al., 2014; Palazova et al., 2013; Syssau et al., 2021), and sensory experience ratings (Hinojosa et al. 2016b; Syssau et al., 2021), we will explore their relationship with positive emotions. Overall, current norms will allow us to further extend our understanding about the influence of positive emotions at different linguistic levels by providing data for a large pool of words in a large set of discrete positive emotions.

## Method

### Participants

A total of 3437 participants (83.50% females, 16.50% males) took part in the study. They completed 5521 questionnaires. Their mean age was 28.55 (SD = 11.71), ranging from 18 to 82 years. They were recruited from different universities covering different areas of the Spanish territory: 54.6% from the UNED (Universidad Nacional de Educación a Distancia), 15.9% from the Universidad Autónoma de Madrid (UAM), 10.0% from the Universitat Rovira i Virgili (URV), 7.8% from the Universidad Complutense de Madrid (UCM), 4.1% from the Universidade de Santiago de Compostela (USC), and 4.9% from other universities. Students received course credits for their participation. Furthermore, 2.8% of the participants were not university students and did not receive compensation. Each participant had the possibility to fill out more than one questionnaire. Each time they completed a questionnaire, they were assigned a random variable and received a randomized set of words. On average, each participant completed 1.61 questionnaires (SD = 1.03). All the participants volunteered for the study and signed an informed consent form before filling out the questionnaires.

### Materials

The words were selected from prior normative studies that have collected affective ratings for Spanish words, both from a dimensional perspective (Ferré et al., 2012; Guasch et al., 2016; Redondo et al., 2007; Stadthagen-González et al., 2017), and from a discrete-emotions perspective (Ferré et al., 2017; Hinojosa et al., 2016a; Stadthagen-González et al., 2018). We included all the words of the datasets of Ferré et al. (2012, 2017), Guasch et al. (2016), Hinojosa et al. (2016a), and Redondo et al. (2007). After removing duplicate words in different sets of stimuli, 3141 words were retained. We selected an additional set of 5859 words from the normative studies by Stadthagen-González et al. (2017, 2018). The potential utility of the emotional features of words to design studies was taken into account to select stimuli. Therefore, we excluded words with a low frequency of use, conjugated verb forms, inflected words, or proper nouns. The total number of words was 9000.

### Procedure

An online questionnaire in the form of a website created from scratch was used to rate the words. In this system, both the variable and the words to be rated in each questionnaire were chosen at random. That is, each time the questionnaire was accessed, the system randomly chose 250 words from the entire list of 9000 words, as well as one of the seven possible variables. Thus, each participant rated a different set of words.

**Fig. 1:**
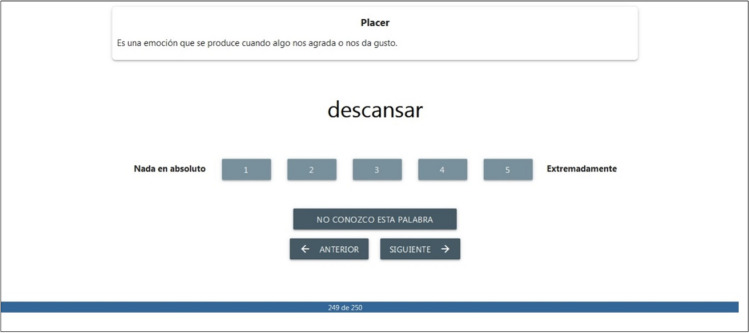
Layout of the rating screen for the word *descansar* (to rest) and the emotion *placer* (pleasure)

When accessing the website, participants first agreed to an informed consent form by ticking a checkbox. Then, they had to provide the demographic information concerning age, sex, and university attended. On the third screen, they were given instructions for the assigned variable in order to rate the words. Participants were asked to rate the extent to which each word conveys an emotion, using a 1 (not at all) to 5 (extremely) scale. Participants were given the name of the emotion, as well as its definition, in the center of the screen. These definitions were adapted from prior normative studies and research aimed at providing taxonomies of positive emotions (Keltner & Cowen, 2021; Shiota et al. 2017; Syssau et al., 2021; Weidman & Tracy, 2020). The exact text of the instructions and definitions of the emotions can be found in the Appendix. After the instructions, participants began to rate the words, which appeared randomly one by one in the center of the screen. There were five buttons under each word with the scale numbers and anchor points at the ends. Participants had the option to say that they did not know the word. The name of the emotion to be rated and its definition appeared at the top of the screen as a reminder (see Fig. 1). Participants took 20 min on average to complete the questionnaire.

### Data trimming and description of de dataset

The 5521 completed questionnaires were submitted to a trimming procedure in order to exclude participants who responded with anomalous patterns. In particular, we discarded questionnaires that were clearly filled out carelessly (e.g., all answers with the same value; 0.60% of the data), questionnaires with more than 25% of answers in the option "I don't know the word" (0.34% of the data), and questionnaires where the scores correlated less than 0.1 with the mean rating of the same words in the rest of the questionnaires of the same variable (2.70% of the data). In addition, questionnaires where the ratings had a standard deviation of more than 2 SD below the mean of the standard deviation of the responses to all words of a given variable (2.66% of the data) were also discarded. For all of these reasons, a total of 348 questionnaires were removed (6.30% of the data), so 5173 questionnaires were analyzed. Each word was rated by 20.53 participants on average (SD = 1.14; range, 20–29). Of note, participants had the option to report that they did not know the word. A total of 6390 words (71% of the total) were not reported to be unknown in any questionnaire, and only 951 words (10.57% of the total) were reported as unknown more than five times (it should be noted that each word was rated by 140 or more participants across variables).

The final database is available at https://doi.org/10.6084/m9.figshare.21533571.v2. It takes the form of a spreadsheet where the following information is included for each word: mean rating, standard deviation, number of raters, and proportion of participants who reported not knowing the word, for each of the seven positive emotions (awe, relief, excitement, amusement, pleasure, contentment, and serenity). Of note, data are not presented split by sex due to the disproportion in the number of males and females in the sample (only 14.15% males in the final set of participants).

## Results

### Validity of the norms

Due to the procedure used, it was not possible to compute inter-rater reliability for these data. The reason for this is that each participant was presented with a random selection of words, so that all questionnaires were different. This drawback is compensated for by the highly randomized presentation of the stimuli. This procedure allows to control the potential word order and list composition effects. Nonetheless, we sampled 250 words from our original pool that were randomly selected for each discrete positive emotion in order to get an estimation of the inter-rater reliability following the standard procedure in normative studies collecting psycholinguistic and affective ratings (e.g., Ćoso et al., 2019, in press; Ferré et al., 2017; Hinojosa et al., 2016a, b; Monnier & Syssau, 2013; Moors et al., 2013; Montefinese et al., 2014; Stadthagen-González et al., 2018). Therefore, we created seven different questionnaires that included 250 different words. Each questionnaire was filled out by 20 individuals who had not participated in the previous collection of data for a total sample of 140 participants (M_age_ = 22.34; SD_age_ = 9.01; 110 females). In this way, we were able to calculate the inter-rater reliability of each questionnaire using the split-half method running 100 sampled iterations corrected with the Spearman–Brown formula. The results yielded high and positive correlation values for all positive emotions: *r* = .90 for awe and relief, *r* = .91 for excitement and serenity, and *r* = .92 for amusement, contentment, and pleasure. Furthermore, we were able to examine the consistency of the scores across different assessments by comparing ratings from the normative study with those that were collected to make an estimation of the inter-rater reliability. Correlations were also high and positive: *r* = .84 for awe, *r* = .88 for relief, *r* = .89 for excitement and contentment, *r* = .90 for amusement and serenity, and *r = *.91 for pleasure (all *p* values < .001).

Finally, to assess the validity of our norms, we relied on the study of Syssau et al. (2021), with which we had five discrete positive emotions in common (awe, excitement, amusement, contentment, and serenity). To look for overlapping words, we subjected the words to a Spanish–French cross-translation using an automatic translator, leaving only those words for which the translator gave the same result in both translation directions. A match was found for 525 words (about half of the words contained in Syssau et al., 2021). The Pearson correlations between databases were *r* = .72 for awe, *r* = .71 for excitement, *r* = .79 for amusement, *r* = .83 for contentment, and *r* = .84 for serenity (all *p* values < .001).

### Descriptive statistics

Table 1 shows the scale, the mean rating, standard deviation, and minimum and maximum values for each of the seven assessed dimensions. The table also includes the same information for the other variables considered in the analyses performed here. The data for happiness, anger, sadness, fear, and disgust were obtained from the Spanish datasets on discrete emotions (i.e., Ferré et al., 2017; Hinojosa et al., 2016a; Stadthagen-González et al. 2018). Valence and arousal values were obtained from Hinojosa et al. (2016a), Stadthagen-González et al. (2017), Guasch et al. (2016), and Ferré et al. (2012). The frequency data (Zipf; subtitle tokens) were extracted from EsPal (Duchon et al., 2013). For all these variables, values were obtained for the entire set of 9000 Spanish words. Furthermore, we obtained concreteness ratings for 5701 Spanish words and familiarity ratings for 5690 Spanish words, using the EsPal database (Duchon et al., 2013), and the databases by Hinojosa et al. (2016b), Guasch et al. (2016), and Ferré et al. (2012). The values from Hinojosa et al. (2016b) and Ferré et al. (2012) were retrieved using EmoFinder (Fraga et al., 2018) because in this application the ratings are converted to the same scale to the rest of the bases (i.e., 1–7). Scores of the age of acquisition (AoA) were taken from Alonso et al. (2015) and Hinojosa et al. (2016b), for 4875 Spanish words. Finally, sensory experience ratings (SER) were retrieved from Díez-Álamo et al. (2019) and Hinojosa et al. (2016b), for 4437 Spanish words.

**Table 1:** Descriptive statistics of the discrete positive emotions, other discrete emotions, dimensional ratings, and other relevant psycholinguistic indices for the 9000 Spanish words

Variable	Rating scale*	Mean	SD	Minimum	Maximum
Awe	1–5	2.30	0.73	1.00	4.95
Relief	1–5	2.30	0.75	1.00	5.00
Excitement	1–5	2.30	0.75	1.00	4.82
Amusement	1–5	2.15	0.71	1.00	4.91
Pleasure	1–5	2.38	0.81	1.00	4.95
Contentment	1–5	2.43	0.84	1.00	5.00
Serenity	1–5	2.28	0.73	1.00	5.00
Happiness	1–5	2.16	0.85	1.00	4.97
Anger	1–5	1.67	0.68	1.00	4.87
Sadness	1–5	1.69	0.74	1.00	4.93
Fear	1–5	1.73	0.71	1.00	4.80
Disgust	1–5	1.55	0.59	1.00	4.83
Valence	1–9	5.19	1.49	1.10	8.85
Arousal	1–9	5.37	1.00	1.40	8.45
Zipf	-	3.52	0.83	0.33	6.69
Familiarity	1–7	5.09	1.04	1.40	7.00
AoA	1–11	7.16	2.08	1.12	10.94
Concreteness	1–7	4.63	0.98	1.99	7.00
SER	1–7	3.70	0.86	1.48	6.54

* For the discrete emotions 1 = Nothing at all, 5 = Extremely; for valence 1 = Completely sad, 9 = Completely happy; for arousal 1 = Completely calm, 9 = Completely energized; for familiarity 1 = minimum level, 7 = maximum level; for AoA 1 = less than 2 years, 2 to 20 = the exact age, 11 = 11 years or older; for concreteness 1 = very concrete, 7 = very abstract; for SER 1 = it does not evoke any sensory experience at all, 7 = it evokes a very strong sensory experience.

### Relationship between discrete positive emotions, negative discrete emotions, affective dimensions, and other psycholinguistic variables

**Fig. 2:**
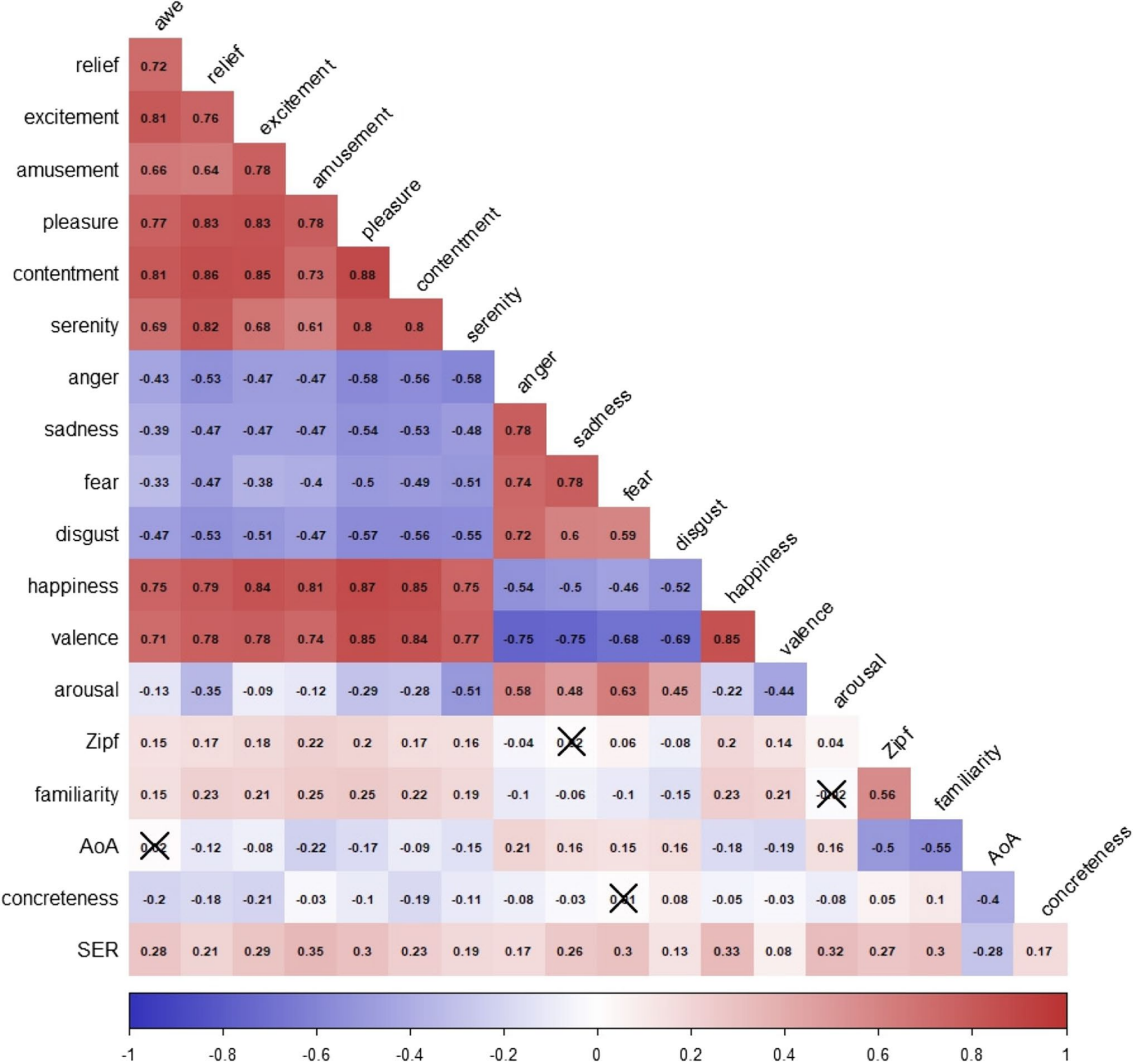
Correlogram between discrete emotion ratings, dimensional ratings, and other psycholinguistic variables. *Crossed out values* indicate non-significant values at a significance level of .05. All *N* = 9,000, except for familiarity (*N* = 5690), AoA (*N* = 4875), concreteness (*N* = 5701), and SER (*N* = 4437)

The correlations between all the variables considered in this study are represented in Fig. 2. First, we examined the pattern of correlations between the seven discrete positive emotions and happiness. These correlations ranged from .61 (serenity – amusement) to .88 (pleasure – contentment).

Concerning the correlation between the discrete positive and negative emotions, the correlations were all negative, as expected (i.e., the higher the rating for a positive emotion, the lower the rating for a negative one). The values ranged from – .33 (awe - fear) to – .58 (anger - pleasure and anger - serenity), all correlations being lower than those found among the positive emotions themselves.

We examined more in depth the relationship between the seven positive emotions and the emotion of happiness by conducting a multiple linear regression where the seven positive emotions were included as predictors, with happiness as the dependent variable. To deal with the multicollinearity of the predictors (the seven variables were highly correlated), we examined the relative contribution of each predictor using the metric *lmg* in the R package *relaimpo* (Grömping, 2006). It provides an average of the *R*^2^ for each predictor in all possible orders in the regression model. After estimation, the resulting model explained 83.43% of the variance. We examined the relative contribution of each emotion to the model, using bootstrapped confidence intervals to assess the significance of the differences. We observed that pleasure was the emotion that contributed the most to happiness in relative terms (18.12% of the variance explained by the model), with a significant difference with respect to the rest. Next, amusement (16.17%), contentment (15.85%), and excitement (15.43%), with no significant differences between them, were followed by relief (12.67%), which differed significantly from the previous group and the next. The last group included awe (10.92%) and serenity (10.84%), which were the emotions that contributed least (but with non-negligible values) to happiness, and with no significant differences between them.

We also examined the correlations between the ratings in discrete positive emotions and those in the affective dimensions of valence and arousal. For valence, all correlations were high and positive (between .71 and .85). In contrast, the correlations between the ratings of the positive emotions and arousal were all negative, the highest being for serenity (– .51) and relief (– .35) and the lowest negative for excitement (– .09) and amusement (– .12).

Regarding the correlation with non-affective variables, word frequency (Zipf) and word familiarity show similar positive correlations with positive emotions, although familiarity correlations are slightly higher, ranging from .15 to .25. This indicates that there is a tendency for more emotionally charged words to be more frequent and familiar to participants. In contrast, the age at which the words are acquired tends to maintain a negative relationship: the more emotionally charged the words are, the earlier the words are acquired. However, the values are generally low, ranging from .08 to .22, and no significant correlation was observed for awe. Concreteness showed a negative relationship with positive emotions, (i.e., the higher the emotional load, the lower the concreteness). Finally, the positive correlations between all positive emotions and SERs indicate that words conveying more positive emotions evoke more vivid perceptual and sensory experiences.

### Distribution of words in the seven positive emotions

We examined the distribution of the words in the seven positive emotions. To that end, we used the same criterion as in previous studies (e.g., Ćoso et al., 2019; Stadthagen-González et al., 2018; Syssau et al., 2021), considering that a word was highly related to a certain emotion if its rating was equal to or greater than 3 (the midpoint of our scale) on that emotion. Hence, words with values below 3 for all emotions might be regarded as having a weak association with specific positive emotions. In addition, following the nomenclature used in those previous studies, words with a value equal to or greater than 3 in only one emotion were considered as "pure" words with respect to that emotion, while words with a value of 3 or higher in more than one emotion were considered as emotionally "mixed". For classification purposes, mixed words were assigned to the emotion in which their rating was higher. In the case of a tie, that word was assigned to all tied categories.

Of the 9000 words studied, 3517 (39.07%) were related to one positive emotion or more. Table 2 shows the number of words that are related to each emotion according to the above criteria, together with the percentage that they represent in relation to the total dataset. The percentage of "pure" words relative to the number of words related to each emotion is also represented in the table. Finally, the average ratings for the target emotion, as well as for valence and arousal, are also included. Contentment is the emotion with more words (955), followed by pleasure (596). The remaining emotions have between 485 (awe) and 366 (amusement) words. Regarding the number of "pure" words (885; 25.16% of the positive words), contentment is the emotion with the lowest percentage of “pure” words (18.12%), and it is the emotion that has the highest number of related words in the dataset. In contrast, amusement, which has the lowest number of related words in the database, is the emotion with the highest percentage of pure words (37.70%).

**Table 2:** Distribution of words in the seven positive categories, percentage of "pure" words in relation to the number of words in their category, and mean ratings in the emotion, valence, and arousal

Emotion	*N*	%	“Pure” %	Mean	Valence	Arousal
Awe	485	5.39	26.39	3.58 (0.42)	6.21 (0.92)	5.38 (0.79)
Relief	395	4.39	24.56	3.62 (0.46)	6.31 (0.90)	4.70 (0.93)
Excitement	425	4.72	20.94	3.61 (0.45)	6.44 (0.83)	5.88 (0.83)
Amusement	366	4.07	37.70	3.64 (0.51)	6.36 (0.97)	5.60 (0.91)
Pleasure	596	6.62	23.49	3.64 (0.48)	6.66 (0.84)	5.08 (1.09)
Contentment	955	10.61	18.12	3.69 (0.46)	6.55 (0.82)	5.22 (0.85)
Serenity	460	5.11	26.09	3.59 (0.43)	6.26 (0.81)	4.19 (0.81)

*N* = number of words in the category; % = percentage of words related to an emotion with respect to the total database; “pure” % = percentage of the words in the category that can be considered as “pure”; Mean = mean rating in the emotion. Valence = mean rating in valence; Arousal = mean rating in arousal. Standard deviations are in parenthesis

We also examined emotionally mixed words to see whether there was any pattern of relationship between the words. The number of emotionally "mixed" positive words (2632; 74.84% of the words with a score equal to or above 3 in at least one positive emotion) clearly outnumbered the "pure" ones. Moreover, typically, those words contained mixtures of several emotions. Only 20.14% of the "mixed" words showed scores equal to or above three in two emotions. The remaining mixed words had high scores in three (15.77%), four (17.78%), five (15.73%), six (16.41%), and even all seven emotions at once (14.17%). For example, words such as *artista* (artist) or *chocolate* (chocolate) had a rating equal to or above three in the seven emotions.

**Fig. 3:**
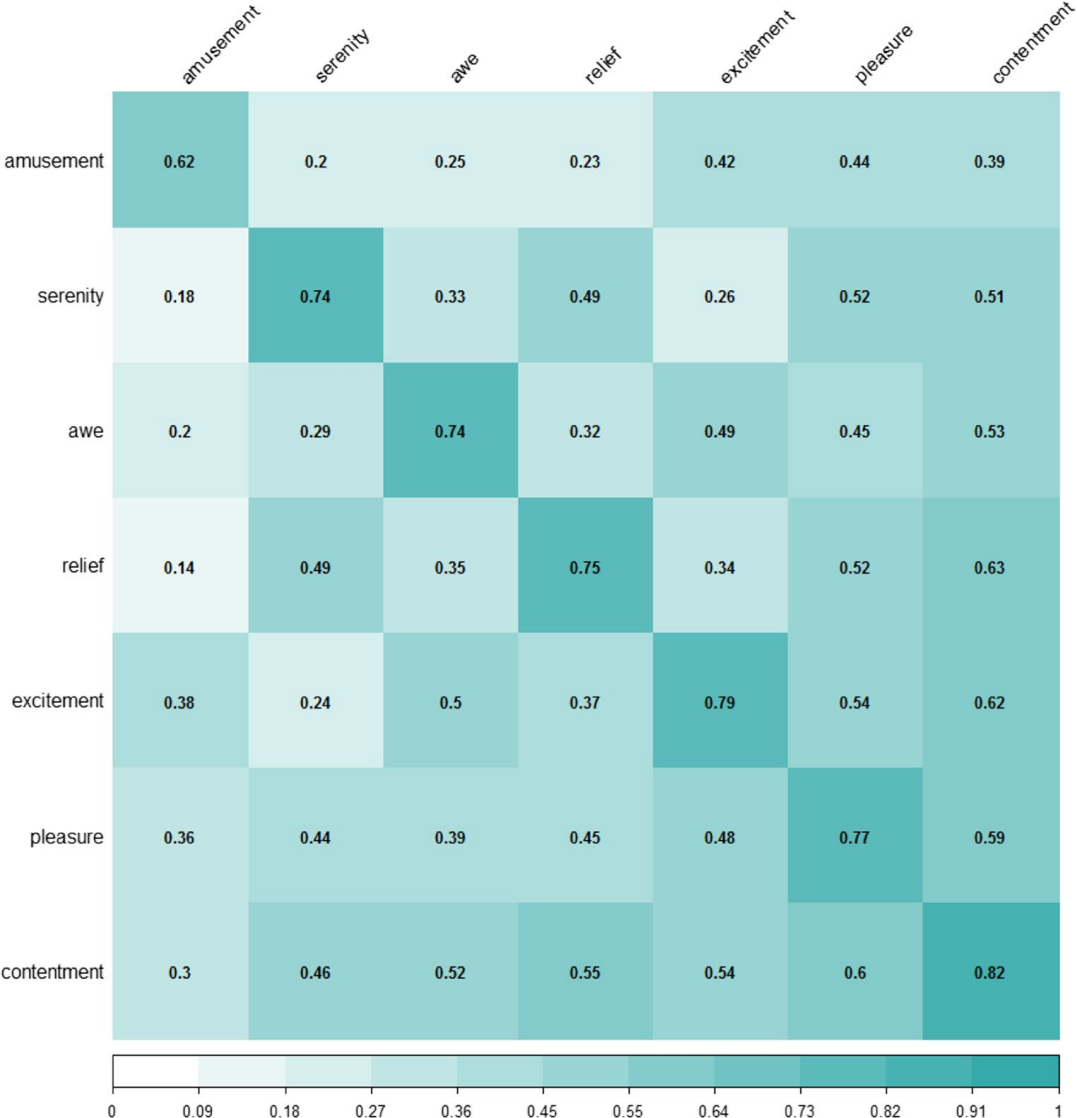
Heatmap depicting the pattern of mixing between positive emotions. Diagonal shows the proportion of words considered "mixed" for that emotion. Each row refers to the words with the highest score in a particular emotion (e.g., contentment in the last row). *Numbers* indicate the proportion of words which are mixed with other emotions. Note that the sum of the proportions (e.g., 0.3, 0.46, 0.52, 0.55, 0.54, and 0.6) is higher than the proportion of mixed words (0.82). The reason is that words can score high in two, three, four or even more emotions

To examine if there was any pattern in the clustering of emotions, we made groups of the mixed words according to the emotion in which they had the highest score. Then we computed the proportion of words in each group that was mixed with the other emotional categories. The result is shown in Fig. 3. As can be seen in the last row, 82% of the words whose highest rating was for contentment showed scores equal to or above 3 in other positive emotions. Among them, pleasure was the emotion with the highest overlap (60% of the words) and amusement the emotion with the lowest overlap (30% of the words).

**Fig. 4:**
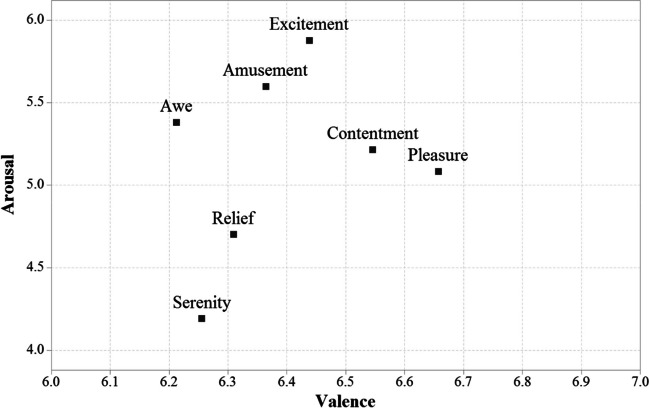
The graph depicts the relationship between mean word ratings for each discrete positive emotion and mean scores in the dimensions of valence and arousal

Finally, we also compared the ratings in valence and arousal of the words related to each emotion with a one-way ANOVA. There were clearly significant differences between conditions for both variables; Valence: *F*(6, 3675) = 19.68, *p* < .001, *η*^2^_p_ = .03; Arousal: *F*(6, 3675) = 173.27, *p* < .001, *η*^2^_p_ = .22. The results are displayed in Fig. 4.

Bonferroni-corrected comparisons for valence revealed that awe, serenity, relief, and amusement did not differ significantly in this dimension (all *p* values > .05). Excitement did not differ from relief, amusement, and contentment (all *p* values > .05) in valence. There were no differences in valence between contentment and excitement or pleasure. Finally, pleasure did not differ from contentment in valence (all *p* values > .05). Concerning arousal, all differences between emotions were significant (all *p* values < .019), except for the comparison between pleasure and contentment, where arousal was the same (*p* = .093).

### The big picture

This study presents normative data for words in seven positive emotions. Interestingly, the 9000 words included here were rated in four negative emotions in previous studies (Ferré et al., 2017; Hinojosa et al., 2016a; Stadhagen-González et al., 2018). This allowed us to examine the distribution of words in both emotional poles to get a more comprehensive view of the characterization of words in terms of discrete emotions. Therefore, we took into consideration the ratings of the words in 11 discrete emotions. We considered as neutral the words whose ratings in all the emotions were lower than 3. The remaining words were classified as belonging to the emotion in which they had the highest score. Words with high scores only in positive emotions were considered as positive words, while words with high scores only in negative emotions were considered as negative words. Following these criteria, we identified a total of 3477 positive words (38.63% of the database), 1254 negative words (13.93% of the dataset), and 4229 neutral words (46.99% of the dataset). There were only 40 words that did not fit in any of the three categories because they had, at the same time, high scores in both positive and negative emotions. Although this may seem counter-intuitive, a close look at these words shows that most of them are emotionally ambiguous. For example, *recuerdos* (memories) is a word with high values for all positive emotions, but also for sadness. Other examples are *león* (lion), which elicits both admiration and fear, *parto* (childbirth), which is related to several positive emotions but also to fear, or *gritar* (to scream), which serves to express both excitement and anger.

**Fig. 5:**
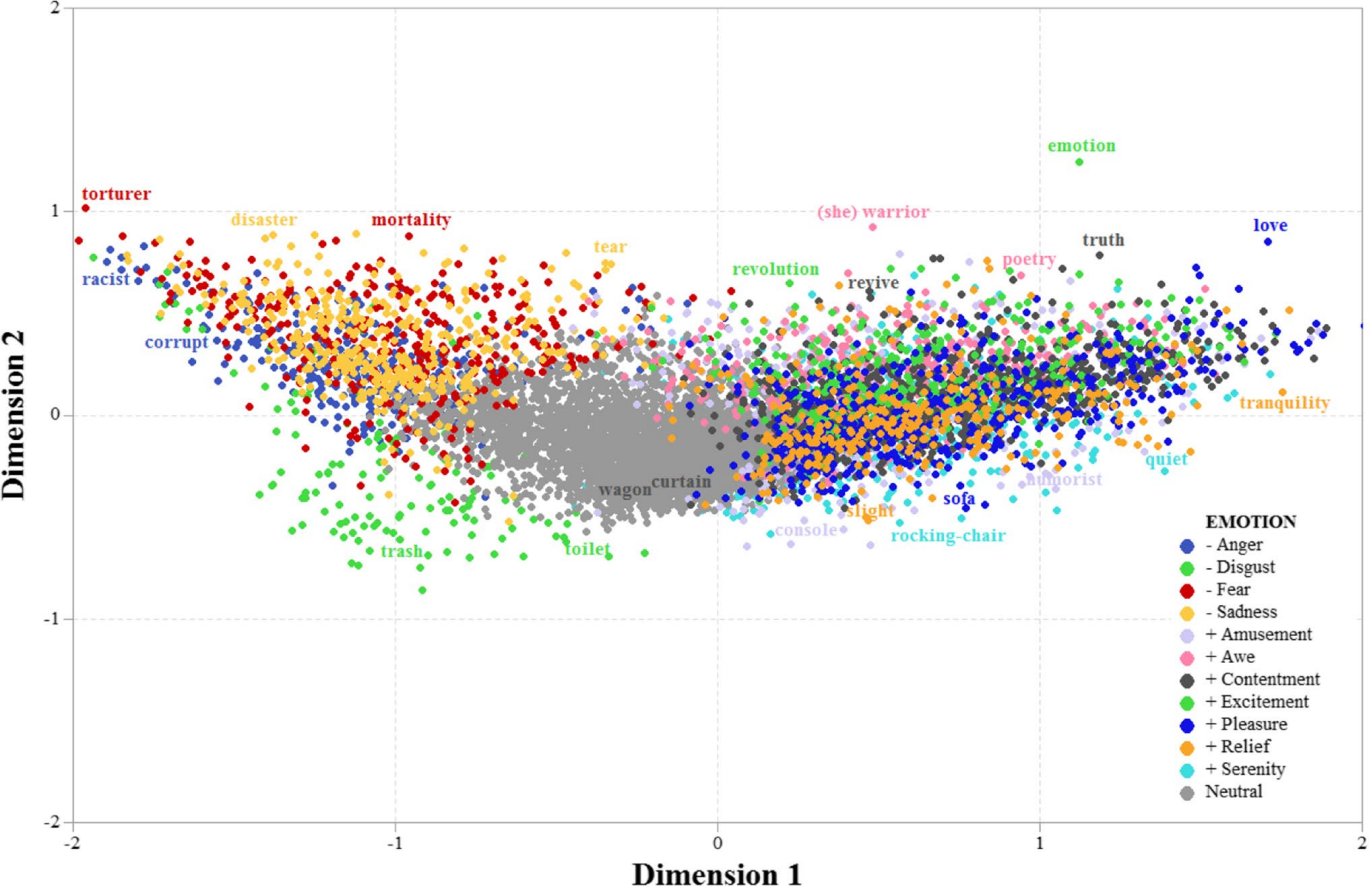
Scatterplot of the coordinates resulting from the MDS. Two example words are included for each emotion

To get a global picture of words’ distribution, we removed the 40 emotionally ambiguous words, and those that showed a tie for the highest score (only 192 words). Then, we submitted the remaining 8768 words to a multidimensional scaling procedure. The advantage of such an approach is that, considering the 11 ratings as a vector of coordinates in an 11-dimensional space, the Euclidean distance between any pair of points can be calculated. Then, a MDS algorithm can be used with this matrix of distances, to make a lower *n*-dimensional projection that preserves as much as possible the distance relations between all the elements. The algorithm employed here was a non-metric MDS algorithm: SMACOF (De Leeuw, 1977; De Leeuw & Heiser, 1977), using the *smacof* package (de Leeuw & Mair, 2009; Mair et al., 2022) in R (R Core Team, 2021). A two-dimensional solution on the data yielded a stress-1 value (a common measure of goodness of fit in MDS) of .081. Considering the low number of dimensions and the high number of points, this fit value can be considered as good. Figure 5 shows a two-dimensional projection of the result of applying this procedure.

In an MDS plot, the dimensions are not directly interpretable because they are a reduction of a larger number of dimensions. However, the distribution of the cloud of points strongly resembled the typical boomerang shape commonly observed in studies relating valence and arousal (e.g., Guasch et al., 2017; Montefinese et al. 2014; Warriner et al., 2013). Regarding the *x*-axis, all negative words were placed on the left side of the graph, neutral words (in grey) in the center, and positive words on the right side. In relation to the *y*-axis, the distribution of words also resembled the typical distribution of arousal, where neutral words tend to have lower values of arousal than negative and positive words. Interestingly, the distribution of positive emotions in the vertical dimension indicates a clear variability, with emotions such as serenity at the bottom of the space, and excitement at its top. Given this resemblance, we performed a Pearson correlation between the values of the coordinates of each dimension, and the ratings in valence and arousal of the words. For valence, the correlation was fairly high (i.e., *r* = .93), suggesting that values in Dimension 1 are probably good indicators of valence. However, for arousal, the correlation was lower (*r* = .52), which indicates that arousal only partially explain the variation on the vertical axis.

## Discussion

Research in affective sciences has expanded to investigate more fine-grained positive emotions that go beyond the broad construct of happiness (Cordaro et al., 2016; Shiota et al., 2017). These studies rely on the availability of large sets of stimuli scored in several discrete positive emotions. However, normative studies on this topic are scarce. Here, we provide norms for 9000 Spanish words for the seven discrete positive emotions of awe, amusement, contentment, excitement, pleasure, relief, and serenity. Current data extend previous normative studies (e.g., Syssau et al., 2021) by reporting scores in a new language (i.e., Spanish), on more positive discrete emotions (ratings for relief and pleasure/liking are collected for the first time), and for a larger set of words. We found that French ratings for awe, amusement, contentment, excitement, and serenity (Syssau et al., 2021) generalize to those of our study, as evidenced by the high correlation between scores. This finding agrees with the observation of cultural, structural, and typological similarities between languages in the representation of affective concepts (Cordaro et al., 2016; Jackson et al., 2019; Lamprinidis et al., 2021), and indicates that ratings for discrete positive emotions are consistent across languages. Similar observations have been reported for words denoting discrete negative emotions such as fear, disgust, anger, or sadness (Ćoso et al., 2022; Stadhagen-González et al., 2018).

Most “pure” words in our dataset denoted amusement (e.g., *discoteca*, discotheque; *cómico*, funny), followed by pleasure and awe. Approximately one-third of the words (3517) in our dataset were scored high in at least one discrete positive emotion. There were 2632 “mixed” words with ratings higher than 3 in at least two discrete positive emotions. We did not observe a clear pattern for “mixed” words since scores overlapped between several, or even all positive discrete emotions. Of note, contentment was the emotion more often “mixed” with other positive emotions. This close relationship between the feeling of perceived completeness and other discrete positive emotions can be accounted for by the role of contentment in supporting well-being, life satisfaction, and skill-building (Fredrickson and Branigan, 2005; Jackson, 2002). These fundamental aspects to physical survival might be core features for the characterization of a broad array of pleasurable states (Fredrickson, 2001). In contrast, serenity showed the lowest overlap with other positive emotions. In line with this observation, there is evidence indicating that serenity is part of a cluster of positive emotions related to the feeling of harmony with their own brain mechanisms (e.g., alpha band power over parieto-occipital regions), whereas positive emotions like amusement or awe are integrated in clusters associated with playfulness and encouragement, respectively (Hu et al., 2017). All in all, the existence of “mixed” words indicates that lexical items can be linked to more than one positive emotion, which possibly depends on pragmatic and contextual cues (Ćoso et al., 2022; Hoemann et al., 2017). Regarding the correlations among discrete positive emotions, and paralleling the findings from our analyses of “mixed” words and correlational analyses with French words (Syssau et al., 2021), it was observed that contentment (like in words such as libre, free; logro, achievement) showed the closest relationship with all discrete positive emotions apart from amusement and happiness. This is also in line with prior claims that suggest that positive emotions such as awe, excitement, or pleasure, might be experienced concurrently with contentment (Cordaro et al., 2016). Additionally, we observed that the emotion elicited by present or expected hedonic events (i.e., pleasure, like in words such as orgasm, orgasm; beso, kiss) is a crucial ingredient of happiness, a blanket term used to describe a broader set of pleasant states. Pleasure is thought to promote activities that lead to survival such as feeding, procreation, or establishing social ties (Alexander et al., 2021; Berridge & Kringelbach, 2011, 2013). These pursuits are related to core aspects of the adaptive function of positive emotions, which might explain our finding of a tight link between pleasure and the basic emotion of happiness. In contrast, serenity (like in words such as agradable, nice; confianza, trust) made the lowest contribution to happiness, and showed the weakest correlations with most discrete positive emotions, including awe, amusement, contentment, excitement, and happiness. This result goes along the same lines of the study by Roth and Lairetier (2021), who asked participants about the intensity with which they had experienced ten positive emotions within the last 48 h. The authors reported that the feeling of inner peace (i.e., serenity) showed a low correlation with all measured emotions, including awe and amusement. Prior normative studies reported a negative correlation between scores in happiness (Hinojosa et al., 2016a; Stadhagen-González et al., 2018), or some discrete positive emotions (Syssau et al., 2021), and ratings in discrete negative emotions like anger, disgust, fear, or sadness. Of note, disgust systematically showed the highest negative correlations with all discrete positive emotions, which suggests that the feeling of aversion to potentially offensive substances or immoral behaviors (Schaich Borg et al., 2008) is perceived as their most antagonist negative emotion (see also Syssau et al., 2021 for similar results).

We also found a pattern of associations between the affective dimensions of valence and arousal, and discrete positive emotions. As expected, all positive emotions were highly correlated with valence. In line with prior reports (Syssau et al., 2021), pleasure-related (e.g., *disfrutar*, enjoy; *cariño*, affection) and contentment-related words (e.g., *sonrisa*, smile; *bienestar*, well-being) were more tightly associated with valence, whereas awe-related words displayed the lowest correlation. In contrast, a negative correlation was observed between all discrete positive emotions and arousal, indicating that words that scored higher in all positive emotions (e.g., *amor*, love; *amistad*, friendship) were those with lower scores in arousal. Similarly, negative correlations between happiness and arousal have been observed in prior normative studies with words (Hinojosa et al., 2016a; Stadhagen-Gonzalez et al., 2018). Also, negative correlations between arousal and both awe and serenity were reported in a study in which participants assessed excerpts of film clips (Hu et al., 2017). However, our results are at odds with those from Syssau et al. (2021), who found that French words with the highest ratings in awe, amusement, contentment, happiness, excitement, and serenity also showed the highest scores in arousal. This divergence might reflect differences in the characteristics of the words used in these two normative studies. In this regard, there were important differences in the number of words (1031 in Syssau et al. vs. 9000 in the current study), the lowest value in arousal (2.14 vs. 1.4), the mean arousal of the words (4.19 vs. 5.37), or the proportion of neutral words (about 7% more neutral words in Syssau et al. relative to the current database). It is also possible that some cross-cultural differences exist in the assessment of arousal. In fact, recent evidence has shown variations in the relationship between valence and arousal in 33 societies with 25 different languages (Yik et al., 2022).

The observations that words conveying a positive response to novelty and new challenges (i.e., excitement-related words) showed a very weak (*r = *– .09), although significant, correlation with arousal deserves further consideration. At first glance, excitement and arousal might be thought of as being closely related concepts. However, a close look to stimuli in our dataset that received the lowest scores in excitement (< 1.5) reveals that they were mainly negative arousing words (e.g., *terrorismo*, terrorism; *violador*, rapist; *humillación*, humiliation). In contrast, those few words that were more closely associated with excitement (> 4.5) show mid to high arousal ratings and positive valence (e.g., *ilusión*, hope; *triunfo*, triumph). Thus, the highest number of negative high-arousing words showing low excitement compared to positive high-arousing words showing high excitement might have biased the relationship between excitement and arousal towards a negative correlation in the current norms^3^.

The relationship between affective dimensions and discrete positive emotions was further examined by analyzing differences in valence and arousal scores for pure words belonging to each discrete positive emotion. Taken together, awe, serenity, relief, and amusement were the positive emotions with the lowest valence scores, although they differed from each other in terms of arousal, with serenity (e.g., *amanecer*, sunrise; *armonía*, harmony) and relief (e.g., *paz*, peace; *descanso*, rest) being the most relaxing emotions. Excitement (e.g., *apasionante*, thrilling; *magnífico*, magnificent) was the emotion with the highest arousal ratings, with an intermediate level of valence, like amusement and contentment. Finally, contentment (e.g., *libertad*, freedom; *amado*, loved) and pleasure (e.g., *caricia*, caress; *música*, music) had an intermediate degree of arousal, and the highest valence scores. Of note, these two emotions cannot be distinguished from each other on the basis of valence and arousal alone. This complex pattern of results suggests that affective dimensions do not fully account for the complex picture of discrete positive emotions since each of them show an idiosyncratic association with valence and/or arousal. In this vein, Hu et al. (2017) used a video-watching paradigm to show differences in brain oscillations for clips eliciting awe, amusement, and serenity, which were thought to arise from a distinct contribution of valence and arousal to each positive emotion. Also, amusement, contentment, pleasure, and relief showed differences in several measures that reflected the activation of several components of the autonomic nervous system activity (e.g., increased heart rate for pleasure, but not for amusement, contentment, or relief; Kreibig, 2010).

Additionally, to explore the distribution of words in the database, we ran multidimensional scaling analyses for those stimuli showing high scores in any discrete positive or discrete negative emotions. The results yielded two dimensions that roughly corresponded to valence and arousal. Of note, Dimension 1 was strongly associated with valence, with negative and positive words located at the left and the right of the graph, respectively. However, the link between arousal and Dimension 2 was weaker since positive and negative words were not always placed at upper parts of this dimension. In this vein, although positive words denoting excitement (e.g., *entusiasmo*, enthusiasm; *emocionate*, exciting) and pleasure (e.g., *pasión*, passion; *deseo*, wish), as well as negative fear-related and anger-related words, were placed at the top of Dimension 2, positive words expressing serenity (e.g., *tranquilidad*, tranquility; *relajación*, relaxation) and relief (e.g., *liberación*, release; *salvación*, salvation) and negative words conveying disgust were placed at the bottom. Thus, Dimension 2 seems not only associated with arousal but also accounts for the variability of discrete positive (and negative) emotions. These findings replicate prior observations (Ćoso et al., 2022) and, once again, suggest that research on emotional language still needs to consider the contribution of both dimensional and discrete approaches to emotions, in line with those views that claim for an integration of both theoretical perspectives (Briesemeister et al., 2014; Harmon-Jones, 2019; Mehu & Scherer, 2015).

An exploration of the correlations between discrete positive emotions and psycholinguistic measures revealed several relationships, in agreement with the results from prior normative (Syssau et al., 2021) and language processing studies (see Citron, 2012 and Hinojosa et al., 2020, for reviews). We observed that all words expressing positive emotions showed higher subjective (i.e., familiarity) and objective frequency of use. A trend towards the use of positive words in written and spoken speech has been observed (Augustine et al., 2011), which possibly arises from the positive connotation of most events in our daily life (Rozin et al., 2010). Also, this positive bias in language use has been observed in the analyses of the most commonly used words in several languages, including English, French, Spanish, or German (Dodds et al., 2015; Kloumann et al., 2012). Of note, our data go further by suggesting that words expressing amusement (e.g., *gracioso*, funny; *chiste*, joke) and pleasure (e.g., *sexo*, sex; *chocolate*, chocolate) are those most frequently used and familiar, whereas awe-related words (e.g., *fascinante*, fascinating; *solidaridad*, solidarity) are used less often.

Prior research has found a positive bias in word acquisition in children (Baron-Cohen et al., 2010; Bahn et al., 2017; Sabater et al., 2022). Similarly, we observed a negative correlation between the age of acquisition and most discrete positive emotions, which indicates that words conveying positive states are learnt earlier in life (see also Ponari et al., 2018; Syssau et al., 2021; Stadhagen-González et al., 2018). This prioritized acquisition of words denoting positive concepts has been related to some features of infant-directed or parentese speech style (Dave et al., 2018). In this sense, based on the analysis of the subcorpus of child-directed speech (MacWhinney, 2000) from the Language Data Exchange System (CHILDES), Ponari et al. (2018) concluded that more than half of the most frequent words in English were positive words. While there is no prior research on the acquisition of words expressing specific positive emotions, our data point to an earlier acquisition of amusement-related words (e.g., *juego*, game; *fiesta*, party), which likely reflects children’s preferences for play and joyful activities (Miller & Kuhaneck, 2008). In contrast, awe-related words showed no correlation with age of acquisition. This finding suggests that core aspects of awe such as perceived vastness and the need for accommodation (Arcangeli et al., 2020; Keltner & Haidt, 2003) are represented by linguist labels conveying feelings of the sublime that can be foreign to children (e.g., *autenticidad*, authenticity; *éxito*, success).

In line with the finding from Syssau et al. (2021), we observed that more abstract words and words with higher SERs were scored higher in all discrete positive emotions. Although correlations were rather low, these data suggest that words conveying positive emotions evoke more vivid multimodal sensory information (e.g., auditory, olfactory, and gustatory information). Also, our findings have some implications for embodied semantic views of emotional words. Based on results from studies showing processing differences between emotional abstract and concrete words (e.g., Ponari et al., 2018; Vigliocco et al., 2014), this perspective assumes a greater degree of affective associations for abstract words (Kousta et al., 2011). Although there is no prior evidence about the role of discrete emotions (either positive or negative) in the processing and representation of abstract words, the observation that excitement showed the highest negative correlation with concreteness indicates that experiences associated with a feeling of novelty and challenge contribute to the semantic representation of abstract words (e.g., *vitalidad*, vitality; *eufórico*, euphoric). In contrast, the negative correlation between amusement and concreteness was almost negligible in the current study, while it was the only discrete positive emotion that had a (weak) positive correlation with concreteness in the study by Syssau et al. (2021). In contrast, this emotion showed the highest positive correlation with SER. Thus, emotions associated with a feeling of perceived humor seem less relevant for embodied abstract semantics, whereas they are associated with a rich repertory of sensory experiences (e.g., *risa*, laughter; *celebración*, celebration). Overall, these findings highlight the complex link between concreteness and SER (Bonin et al., 2018; Syssau et al., 2021) and the need to test the assumptions made by embodied approaches in future studies that investigate the relationship between concreteness or SER and discrete positive emotions.

### Limitations

Our study is not free of some limitations, which should be addressed in future work. First, the set of positive discrete emotions that participants had to assess represented seven out of more than 30 distinct subjective positive emotions that have been examined in prior research (Weidman et al., 2017). Our selection was guided by prior normative studies in French (Syssau et al., 2021), taxonomies or lexically driven classifications of positive emotions (Roseman et al., 1990; Weidman & Tracy, 2020), and those which have received more attention in prior research (e.g., Deutsch et al., 2015; San Martín et al., 2020, for relief; Berridge & Kringelbach, 2013; Sharot et al., 2009, for pleasure). Nonetheless, even though this is up-to-date the largest normative study (in terms of both the number of stimuli and positive emotions), our norms do not cover the full spectrum of positive emotions. Thus, additional efforts are needed to collect ratings of other representative and frequent discrete positive emotions such as pride, hope, compassion, or love.

Another limitation concerns the existence of individual differences in participants’ ratings. Although the inter-rater agreement was rather high in our norms and the average SDs were low for all positive emotions, there were few words that showed high variability in one or several emotions (e.g., *queso*, cheese, SDs = 1.84 and 1.91, for awe and relief, respectively; *hidromasaje*, whirlpool bath, SD = 1.84, for excitement; *cachorro*, puppy, SD = 1.77 for amusement; *universe*, universe, SD = 1.71 for pleasure). These stimuli should be avoided when designing experiments aimed at exploring the influence of a particular positive emotion in word processing. Also, our sample was mainly comprised of young university students, which might not necessarily represent the entire Spanish population. This leaves future work to examine whether scores collected here generalize to individuals from different socioeconomic and cultural backgrounds. Similarly, the characteristics of our sample did not allow one to explore whether demographic factors such as gender or age moderated the assessment of words expressing different positive emotions. Yet, there is evidence indicating gender or age differences in the experience of positive emotions such as gratitude (Allemand & Hill, 2016) or pride (Else-Quest et al., 2012).

Finally, as one reviewer noted, the validity of our norms might be biased by a higher agreement in the ratings for more familiar or frequent words that were more likely to be included as stimuli in both Syssau et al.’s (2021) studies and ours than less common words. A similar potential concern might be expressed regarding the reliability of our norms, which was tested in a random selection of 250 words for each discrete positive emotion. Nonetheless, even though both validity and reliability were examined in a subset of words, the correlations were high (ranging from *r = *.71 to *r = *.92). Moreover, we also observed a high consistency in the scores provided by two independent samples of participants at different times for every positive emotion (ranging from *r = *.84 to *r = *.91). All in all, these findings argue for the generalizability of the norms reported here.

## Conclusion

In recent years, the characterization of several discrete positive emotions with their own neurobiological, behavioral, and psychological mechanisms have challenged mainstream theoretical views in affective science that argued for the existence of a single positive emotion (i.e., happiness). Theoretical efforts have been made to provide a taxonomy of positive emotions that guide research within this emerging field (e.g., Fredrickson, 1998; Keltner & Cowen, 2021; Shiota et al., 2017; Weidman & Tracy, 2020). However, studies that provide stimuli assessed in different discrete positive emotions are scarce. Here we provide scores for a large set of 9000 Spanish words in seven positive emotions. These norms will hopefully allow research into the relationship between positive emotions and language processing.

## Data Availability

Data will be available upon request.

## Appendix

Instructions and definitions in Spanish and their corresponding English Translation

**Note:** for each survey, [EMOCIÓN] was replaced by the corresponding emotion and [DEFINICIÓN EMOCIÓN] was substituted by the corresponding emotion definition: ADMIRACIÓN (Awe), ALIVIO (Relief), DIVERSIÓN (Amusement), ENTUSIASMO (Excitement), PLACER (Pleasure/liking), SERENIDAD (serenity), SATISFACCIÓN (Contentment).

### Instructions

A continuación, se te presentarán una serie de palabras. Te pedimos que valores hasta qué punto cada una de las palabras se relaciona con la emoción de [EMOCIÓN].

[DEFINICIÓN EMOCIÓN]

Para valorar las palabras deberás marcar la puntuación que estimes en una escala de 1 a 5, siendo 1 "nada en absoluto" y 5 "extremadamente".

### Definitions

Admiración: La admiración puede definirse como un estado asociado al asombro y a la contemplación. Cuando alguien siente esta emoción su mente se amplía y se expande la comprensión de lo que le rodea; es un sentimiento que te deja atónito y te hace querer explorar cada detalle de la experiencia.

Satisfacción: La satisfacción se relaciona con un sentimiento de realización personal. Suele aparecer cuando se cubren las necesidades de una persona. Lleva a la persona a sentirse unida con lo que le rodea y le lleva a percibir su entorno como seguro.

Diversión: La diversión es una emoción relacionada con el juego o el humor. Normalmente aparece cuando a alguien le apetece reírse o bromear con otras personas.

Entusiasmo: El entusiasmo tiene que ver con una respuesta positiva a la novedad y a desafíos nuevos. Aparece cuando un objetivo se percibe como interesante y permite a las personas actuar ante los diferentes estímulos que les rodean.

Serenidad: La serenidad es un sentimiento que se puede relacionar con la calma y la tranquilidad. Podemos definir la serenidad como un estado de paz interior, confianza y conexión que es independiente de los acontecimientos externos y que permite disfrutar de las experiencias del presente.

Alivio: Es una emoción relacionada con la seguridad y la relajación que acompañan a la finalización o superación de una situación que nos provoca ansiedad y angustia. Es un sentimiento que surge cuando desaparece la expectativa o la presencia de algún acontecimiento que nos hace sentir incómodos, nos molesta o nos causa dolor.

Placer: Es una emoción que se produce cuando algo nos agrada o nos da gusto.

### Instructions (translation from Spanish)

Next you will see a series of words. We ask you to rate to what extent each of them relates to the emotion of [EMOCIÓN].

[DEFINITION OF EMOCIÓN]

To assess the words, you should give your rating on a scale of 1 to 5, where 1 means “not at all” and 5 “extremely”.

### Definitions (translation from Spanish)

Awe: Awe can be defined as a state associated with amazement and contemplation. When someone feels this emotion, her mind broadens and her understanding of what surrounds her expands; it is a feeling that leaves the person speechless and makes her want to examine everything about her experience.

Contentment: Satisfaction is related to a feeling of personal accomplishment. It brings the person to feel united with what is around her, and to perceive the environment as safe.

Amusement: Amusement is an emotion associated with play or humor. It often appears when someone wants to laugh and joke with other people.

Excitement: Excitement is a positive response to novelty and new challenges. It appears when a goal is perceived as interesting and allows people to react to the different stimuli of the environment.

Serenity: Serenity is a feeling that can be associated with calm and tranquility. We define serenity as a state of inner peace, trust and connection with environment that is independent of external events, and that allows an individual to enjoy her present experiences.

Relief: Relief is an emotion related to a state of security and relaxation following the completion or overcoming of an event that elicits anxiety and distress. It is a feeling that arises when the expectation or the presence of an event that makes us feel uncomfortable, bothers us, or causes us pain disappears.

Pleasure: Pleasure is an emotion elicited by something that pleases us or is joyful.

## Footnotes

1 Some of the conceptual claims made by the basic emotion theory, such as the notion of universality or the existence of coordinated packages of biological markers, have been questioned. Most criticisms come from constructivist approaches, which conceive emotions as conceptual facts (Barret et al., 2007; Barrett, 2011; Clark-Polner et al., 2017). This has generated an intense debate with those who defend the veracity basic emotion model (Izard, 2007; Panksepp, 2007; Scarantino & Griffiths, 2011), which is out of the scope of the current study.

2 The emotion of pleasure/liking might be closely associated with other affective constructs such as happiness or (positive) valence. Nonetheless, there are some critical differences that qualify them to be considered as separate or discrete entities in most classifications of positive emotions. In this line, the expectation of stimuli such as food, sexual orgasm, or drugs, as well as the motivation to get a reward depending on incentive salience of these stimuli, have been highlighted as being idiosyncratic aspects of pleasure/liking. Also, the causation of pleasure/linking strongly relies on the activation of specific subcortical structures, including dopamine circuits in the nucleus accumbens and ventral pallidum. In contrast, happiness has mainly been associated with a feeling of well-being and a transcendent human life goal. The processing of happiness mainly involves the activation of cortical regions such as the orbital frontal cortex or the medial prefrontal cortex. Finally, (positive) valence is a broad concept to encompass all positive experiences and feelings, which is linked to the activation of a wide network of brain regions (Alexander et al., 2021; Berridge & Kringelbach, 2013; Jongbloed & Andrés, 2015; Sharot et al., 2009; Shiota et al., 2017).

3 To test this hypothesis, we ran two separate correlation analyses between excitement and arousal for words that scored high (> 4.5; *n* = 35) and words that scored low (< 1.5; *n* = 1303) in excitement. In line with our prediction, we observed a positive correlation (*r* = .19) for the former and a negative correlation (*r =* – .30) for the later.
